# Traditional Chinese medicine (TCM)-based nanoformulations for cancer therapy: antitumor mechanisms, challenges, and future directions

**DOI:** 10.3389/fphar.2026.1912368

**Published:** 2026-09-28

**Authors:** Xiaoyu Yang, Shuailong Jia, Meiling Wu, Yi Zhou

**Affiliations:** 1 Department of Pharmacy, Union Hospital, Tongji Medical College, Huazhong University of Science and Technology, Wuhan, Hubei, China; 2 Hubei Provincial Clinical Research Center for Precision Medicine for Critical Illness, Wuhan, China; 3 Hubei Key Laboratory of Natural Active Polysaccharides, Union Hospital, Tongji Medical College, Huazhong University of Science and Technology, Wuhan, Hubei, China; 4 Department of Pharmacy, Renmin Hospital of Wuhan University, Wuhan University, Wuhan, China

**Keywords:** anti-tumor treatment, cancer therapy, nanomedicines, nanoparticles, traditional Chinese medicine

## Abstract

**Background/Objectives:**

Traditional Chinese medicine (TCM)-derived compounds have anticancer potential because of their multi-component, multi-target actions and generally favorable toxicity profiles. Clinical translation, however, is limited by poor aqueous solubility, rapid systemic metabolism, and insufficient tumor-selective delivery. This review critically evaluates recent TCM-based nanoformulations developed to address these limitations and improve the therapeutic performance.

**Methods:**

Following the Preferred Reporting Items for Systematic Reviews and Meta-Analyses (PRISMA) and EQUATOR guidance, PubMed and Web of Science databases were searched for original studies published during the previous 5 years using terms including “TCM,” “nanoformulation,” and “anticancer.” After screening 387 records, 81 studies covering 50 representative TCM compounds were included and assessed for the preparation methods, antitumor mechanisms, and translational barriers.

**Results:**

Relative to free compounds, nanoformulations frequently improved solubility, stability, apparent bioavailability, or the targeting efficiency of TCM constituents. Reported mechanisms included apoptosis, cell-cycle arrest, ferroptosis, immunogenic cell death, and tumor-microenvironment remodeling across breast, lung, colorectal, and other cancer models. Nevertheless, most evidence was derived from *in vitro* experiments or small animal studies, and direct cross-study comparison was limited by heterogeneous formulations, doses, models, and endpoints.

**Conclusion:**

TCM-based nanoformulations represent a promising strategy for integrating traditional herbal medicine with modern cancer therapy. Despite these promising findings, substantial challenges remain in standardization, reproducibility, pharmacokinetics, long-term safety, manufacturing, and clinical validation. Future studies should prioritize head-to-head comparisons, quantitative biodistribution, clinically relevant models, and scalable manufacturing.

## Graphical Abstract

**Figure:**
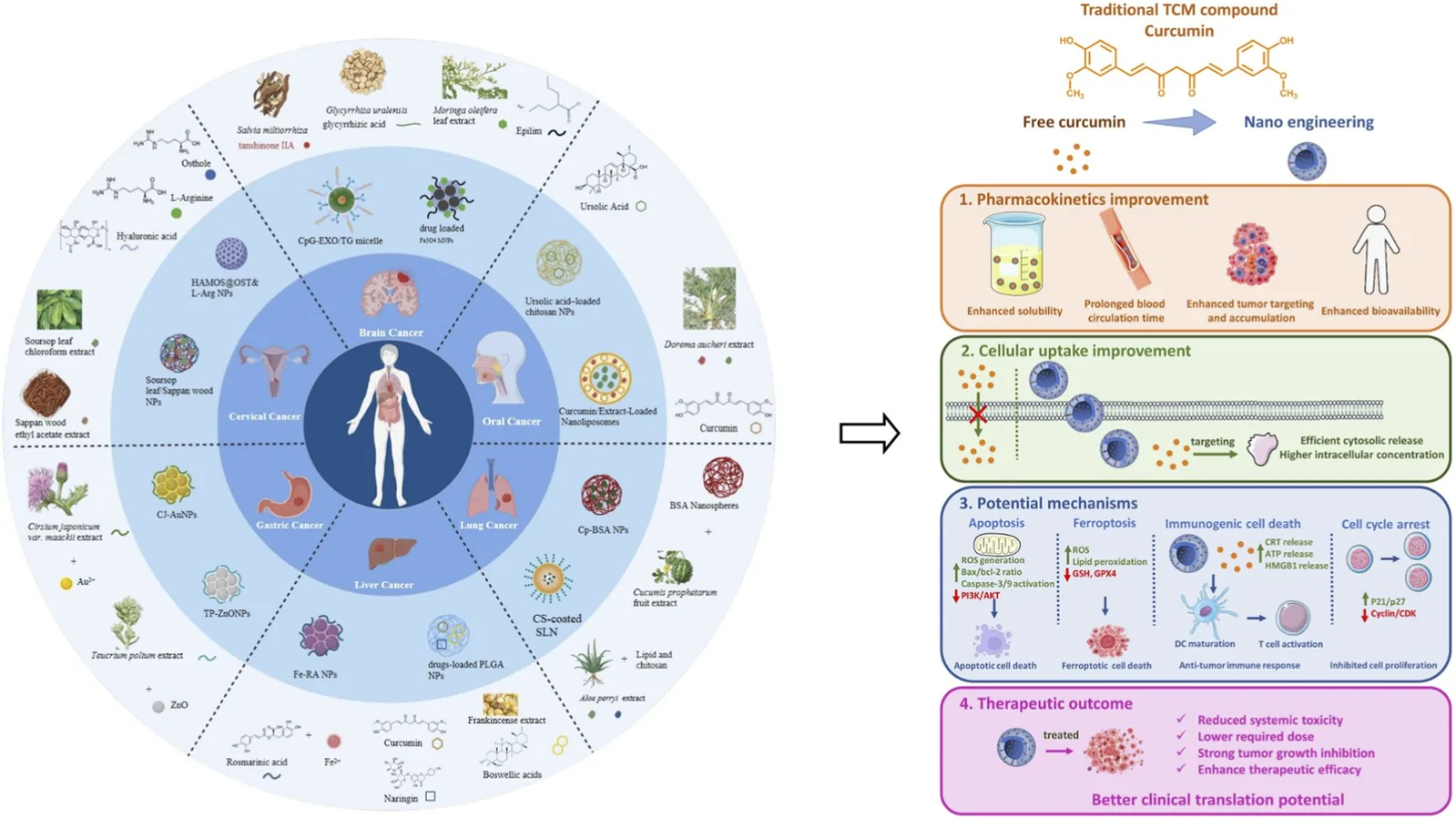

## 1 Introduction

Cancers are major diseases threatening human health, with rising global incidence and mortality. GLOBOCAN 2024 (IARC) reported 20.6 million new cancer cases and 9.8 million deaths worldwide in 2024, with a projected 67% increase by 2050. Currently, clinical tumor treatments mainly rely on surgical resection, chemotherapy, radiotherapy, and molecular targeted therapy (Hnamte and Pulikkal, 2024). However, conventional chemotherapeutic agents generally have poor water solubility, unsatisfactory biodistribution, insufficient targeting, and significant systemic toxicity, which seriously affect the treatment effects and reduce the patients’ quality of life (Abdifetah and Na-Bangchang, 2019). Therefore, the development of new antitumor drug delivery systems that are highly effective and low-toxic has become one of the key research directions.

Traditional Chinese medicine (TCM) has a long history of clinical application in tumor prevention and treatment. Many TCM-derived bioactive compounds have favorable safety profiles compared with conventional chemotherapeutics, although toxicity is compound- and dose-dependent and requires systematic evaluation (Fu et al., 2025). The multi-component and multi-target characteristics of TCM offer distinct advantages by simultaneously modulating tumor proliferation, apoptosis, immune responses, and drug resistance (Zhang et al., 2025). Nevertheless, active constituents of TCM, including flavonoids, alkaloids, saponins, and polysaccharides, generally exhibit limitations such as poor aqueous solubility, chemical instability, rapid metabolism, and inadequate tissue distribution, which reduce their bioavailability and constrain clinical efficacy.

Nanotechnology has emerged as a transformative strategy to overcome the pharmacological bottlenecks of TCM active ingredients. Advanced nano-drug-delivery systems, comprising lipid-based nanocarriers (liposomes, micelles, and nanoemulsions), polymeric nanoparticles (NPs), and inorganic nanomaterials, are capable of effectively encapsulating TCM active ingredients, thereby significantly improving their solubility and stability (Jia et al., 2023). These nano-drug-delivery systems utilize distinct physicochemical properties, including nanoscale size effects and surface engineering capabilities, to optimize drug pharmacokinetics. Specifically, they facilitate passive targeting via the enhanced permeability and retention effect (EPR) and enable active targeting through ligand–receptor interactions, thereby optimizing tumoral drug accumulation while minimizing the side effects. More importantly, these nano-systems enable co-loading of multiple TCM active ingredients with different antitumor mechanisms, realizing synergistic antitumor effects that cannot be achieved by single-drug delivery. In summary, TCM-based nano-delivery systems integrate the theoretical advantages of TCM with modern nanotechnology, providing innovative and promising strategies for precision antitumor treatment and breaking through the limitations of traditional TCM clinical application.

Despite the considerable potential of TCM-based nano-delivery systems in cancer therapy, several critical challenges remain to be addressed for their clinical translation (Islam et al., 2021). The complexity of TCM components and the diverse physicochemical properties of different active ingredients pose difficulties in achieving efficient co-loading, stable release, and synergistic enhancement of therapeutic mechanisms. Additionally, issues such as the scalability of nanocarrier preparation, long-term biosafety, and standardized quality control systems need to be resolved. Specifically, these translational barriers are multifaceted: (1) batch-to-batch variability in herbal extracts—arising from differences in geographical origin, harvest season, and cultivation practices—undermines quality consistency; (2) the precise identification and quantification of bioactive constituents within complex, multicomponent mixtures remain technically challenging, thereby impeding the elucidation of structure–activity relationships; (3) regulatory pathways for TCM-based nanomedicines lack harmonized, science-based guidelines, introducing uncertainty throughout preclinical and clinical development; and (4) a significant efficacy gap exists between robust preclinical findings and modest clinical outcomes—largely attributable to poor formulation reproducibility and interspecies pharmacokinetic disparities—resulting in few candidates progressing to human trials. Addressing these interconnected challenges is indispensable for realizing the therapeutic potential of TCM nanomedicine. Unlike reviews that focus primarily on single-type nanocarriers or single TCM active ingredients, this article systematically reviews the classification, preparation strategies, and antitumor applications of TCM-based nano-delivery systems across various tumor types, as well as their underlying molecular mechanisms of action. Furthermore, we discuss the current challenges and future directions, with the aim of identifying evidence gaps relevant to TCM modernization and precision cancer therapy.

## 2 Literature screening and selection process

To comprehensively elucidate the current advances, underlying antitumor mechanisms, preparation strategies, and clinical translational challenges of TCM nanoformulations in cancer therapy, a systematic literature search and screening procedure was conducted in strict accordance with the PRISMA 2020 guidelines and EQUATOR Network reporting standards.

### 2.1 Databases and time frame

Electronic databases including PubMed, Web of Science, and Scopus were searched for publications from January 2020 to December 2025. Additional references were retrieved by manually screening the bibliographies of relevant reviews and primary articles.

### 2.2 Search strategy

A hybrid search strategy combining Medical Subject Headings (MeSH) terms and free-text keywords was applied. The core search terms covered three major thematic categories: traditional Chinese medicine (“traditional Chinese medicine” OR “herb” OR “TCM active ingredients”), nano-delivery system (“nanoformulation” OR “delivery system” OR “nanocarriers” OR “nanoparticles”), and tumor therapy (“anticancer” OR “antitumor” OR “cancer therapy”). Search terms were logically combined, and the retrieval syntax was appropriately adjusted to adapt to the searching rules of each database.

### 2.3 Eligibility criteria

Uniform eligibility criteria were established to screen qualified literature for this systematic review, with detailed inclusion and exclusion items specified as follows.

#### 2.3.1 Inclusion

i. Original experimental or clinical studies investigating the antitumor properties, fabrication strategies, or translational prospects of TCM-based nanoformulations. ii. Valid *in vitro* cellular experiments, *in vivo* animal model studies, or human clinical trials relevant to TCM nanoformulation cancer therapy. iii. Peer-reviewed English publications with accessible full texts and complete research data.

#### 2.3.2 Exclusion

i. Systematic reviews, editorials, and conference abstracts without original empirical data. ii. Studies merely focusing on TCM ingredients or nanocarriers alone without valid nanoformulation construction and antitumor outcome indicators. iii. Genetic polymorphism studies lacking functional verification of nanoformulation-mediated anticancer effects. iv. Non-English literature and studies with incomplete or unavailable full-text data.

### 2.4 Screening procedure

All the retrieved literature records were standardized screened according to the PRISMA criteria. Two independent reviewers first evaluated all the titles and abstracts to exclude duplicate, irrelevant, and noncompliant studies. Then, the full texts of the candidate articles were evaluated to determine the final inclusion criteria. Any differences that arose during the screening process were resolved through group discussions or consultation with additional reviewers to ensure the accuracy and reproducibility of the screening. Through systematic database search and manual literature supplementation, a total of 387 preliminary records were obtained.

### 2.5 Data extraction and quality assessment

Following comprehensive screening, a final set of 81 eligible studies covering 50 representative TCM compounds was included in this systematic review. For each enrolled study, standardized data extraction and rigorous quality assessment were carried out. We specifically extracted the following core research information: types of TCM compounds and their classified categories; structural characteristics and fabrication modes of TCM nanoformulations; key antitumor signaling pathways and molecular mechanisms. Additionally, qualitative quality evaluation was performed to assess the methodological rigor, data integrity, and experimental reproducibility of all included studies, ensuring high-quality and credible evidence synthesis for summarizing the research progress and translational limitations of TCM nanoformulations in anticancer therapy.

## 3 Preparation strategies of TCM-loaded nanoparticles

TCM-derived constituents are of interest in oncology because they can act on multiple pathways and may have favorable toxicity profiles. However, poor solubility, rapid metabolism, and inadequate tumor exposure restrict their clinical application (Ai et al., 2024; Fu et al., 2025; Yang L. et al., 2024). Nanocarrier design should, therefore, be selected according to payload hydrophilicity, molecular size, chemical stability, desired release profile, route of administration, and the intended biological barrier. Preparation approaches can be grouped into organic, inorganic, and hybrid systems, each with different trade-offs in loading, stability, biodegradability, manufacturability, and safety. No carrier is universally optimal; formulation selection should be driven by a defined therapeutic and translational need rather than by novelty alone.

### 3.1 Organic nanocarriers

For organic nanocarriers, several well-established methods are used because of their biocompatibility and biodegradability. Liposomes, which are spherical vesicles with a phospholipid bilayer, are often prepared using techniques such as film hydration, reverse evaporation, or microfluidic technology. With these methods, hydrophilic TCM constituents are encapsulated in the aqueous core, whereas hydrophobic constituents partition into the lipid bilayer. For instance, baicalin-loaded liposomes prepared by reverse evaporation exhibited enhanced drug solubility and distribution efficiency in the brain (Gu et al., 2024). However, a critical challenge for liposomal formulations is the low encapsulation efficiency observed for TCM compounds with intermediate hydrophilic–lipophilic balance as such compounds tend to partition unfavorably between the aqueous core and the lipid bilayer. Moreover, conventional scale-up synthesis methods can introduce batch-to-batch variability in both particle size and drug loading. Microemulsions and nanoemulsions, which are thermodynamically or kinetically stable dispersions, can be formulated via spontaneous emulsification or high-energy homogenization. These formulations are particularly suitable for oily TCM extracts, such as coix seed oil-based microemulsions, which can be functionalized with transferrin to target cervical cancer cells (Guo et al., 2019). These systems effectively solubilize lipophilic TCMs at high concentrations without chemical modification, but they face critical limitations—microemulsions require cytotoxic surfactant levels, and nanoemulsions are destabilized by Ostwald ripening (particle growth and drug leakage), restricting their chronic clinical use. Polymeric NPs, including those from poly(lactic-co-glycolic acid) (PLGA) or chitosan, are commonly produced by nano-precipitation or emulsion-solvent evaporation. Nano-precipitation involves dissolving TCM and the polymer within a solvent that is miscible with water (e.g., acetone) and adding it drop-wise to an aqueous solution under stirring, causing instantaneous nanoparticle formation (Zielińska et al., 2020). This method is advantageous for its simplicity and low energy consumption, but it is limited to TCM compounds soluble in water-miscible organic solvents, and the residual solvent may pose toxicity risks. The double emulsion (W/O/W) solvent evaporation method is advantageous for hydrophilic TCM macromolecules, such as Chinese yam polysaccharide PLGA nanoparticles (CYPP) with immunomodulatory effects, yet it suffers from low encapsulation efficiency for high-molecular-weight TCMs due to rapid diffusion of hydrophilic molecules into the external aqueous phase (Iqbal et al., 2015). Amphiphilic block copolymers self-assemble into micelles, which are prepared through direct dissolution or dialysis, facilitating the incorporation of hydrophobic TCM drugs such as triptolide or curcumin (Bu et al., 2021). Micelles effectively solubilize poorly water-soluble TCMs with minimal surfactant, but their high critical micelle concentration (CMC) causes rapid dissociation in the bloodstream, lowering drug delivery efficiency. Nanostructured lipid carriers (NLCs) and solid lipid nanoparticles (SLNs) are typically produced using high-pressure homogenization (hot or cold), where the molten lipid phase containing the TCM drug is passed through a narrow gap under high pressure, as applied to artesunate-loaded SLNs for enhanced efficacy against esophageal cancer (Akanda et al., 2023). SLNs are biocompatible and leak-resistant but have limited drug loading owing to their solid matrix. NLCs use liquid lipids to generate matrix defects, boosting loading and stability, yet they necessitate tight temperature regulation to prevent lipid breakdown during processing.

### 3.2 Inorganic nanocarriers

Inorganic nanocarriers involve synthetic routes tailored to their material properties. Mesoporous silica nanoparticles (MSNs) are prepared via a sol–gel process using a silica precursor (e.g., tetraethyl orthosilicate) and a structure-directing template (e.g., cetyltrimethylammonium bromide) under controlled pH and temperature, with template removal creating porous structures for loading TCM drugs such as evodiamine and berberine for pH-responsive combination therapy (Chen J. et al., 2024). Metal NPs such as gold nanoparticles (AuNPs) or iron oxide nanoparticles (IONPs) are synthesized through chemical reduction or co-precipitation. For example, curcumin-conjugated AuNPs were synthesized by reducing chloroauric acid with curcumin and a stabilizer such as polyvinylpyrrolidone (PVP), allowing for pH-sensitive drug release (Hammami et al., 2021). Metal nanoparticles offer compelling advantages including favorable biocompatibility, readily tunable surface chemistry for targeted delivery, and multifunctional imaging potential (e.g., magnetic resonance guidance with IONPs). However, they share critical limitations: elevated manufacturing costs, size-sensitive performance, aggregation during fabrication that compromises reproducibility, and the risk of prolonged retention in reticuloendothelial organs such as the liver and spleen, all of which impede their long-term clinical applicability. Metal–organic frameworks (MOFs), characterized by large surface area, are synthesized by coordination self-assembly of metal ions with organic linkers under solvothermal conditions, enabling high loading of celastrol in ZIF-8 nanoparticles (Zeggai et al., 2025). MOFs achieve the highest drug loading among inorganic carriers, yet they suffer from structural instability in physiological environments (e.g., pH shifts and high ionic strength), causing premature release. Moreover, metal-ion toxicity demands careful scrutiny, which, together with instability, hinders their clinical translation relative to organic carriers.

Post-synthesis functionalization and surface engineering techniques are critical for achieving precise drug release control. Surface modification through polyethylene glycol (PEGylation) confers stealth properties, thereby prolonging blood circulation by reducing opsonization. Targeting ligands such as folic acid, transferrin, or antibodies can be anchored to bind receptors that are overexpressed on cancer cells. This approach is exemplified by folic acid-functionalized MOFs loaded with bufalin (FA-MOF/Buf), which are employed for targeted breast cancer therapy (Singh et al., 2024). While ligand functionalization enhances targeting specificity, it increases the formulation complexity and production costs, and ligand density must be optimized to avoid nonspecific binding. Stimuli-responsive nanocarriers are rationally engineered to achieve tumor microenvironment-specific release of TCM payloads through the incorporation of pH-labile bonds, redox-sensitive linkers, or enzyme-cleavable sequences, typically with functionalized polymers or cross-linkers (Rajankar et al., 2025). A key challenge is off-target stimuli response (e.g., pH shifts in normal tissues), which reduces drug release selectivity and thus demands more precise design of responsive moieties.

Overall, the nanocarrier composition and preparation methods directly affect drug loading, stability, release, biodistribution, and safety (Yang L. et al., 2024). Organic carriers generally offer better biodegradability and a more established translational path, but they may have limited loading or physical stability. Inorganic carriers provide high loading and imaging or catalytic functions, yet they raise greater concerns about persistence, organ accumulation, and long-term toxicity. Hybrid designs may combine these advantages but increase manufacturing and regulatory complexity. Comparative studies should, therefore, use formulation-matched controls and report stability, batch variability, pharmacokinetics, and material fate alongside antitumor efficacy.

## 4 Antitumor applications of TCM nanoformulations

### 4.1 Head and neck cancer

The management of head and neck cancers is confronted with considerable therapeutic obstacles, including anatomical complexity, poor drug bioavailability, and nonspecific toxicity of conventional therapies. This section highlights recent advances in nanoformulations for brain and oral cancers, including magnetic targeting, bio-inspired carriers, and natural compound delivery (shown in Table 1).

**TABLE 1:** TCM nano-formulations for the treatment of head and neck cancer.

TCM/active components of TCM	Types of NPs	Materials of NPs	Types of cancer treated	Years	References
*Moringa oleifera*	Magnetite NPs	Ferriferous oxide	Brain cancer	2024	Waqar et al. (2024)
Tanshinone IIA and glycyrrhizic acid	Micelle	Tanshinone IIA and glycyrrhizic acid	Glioblastoma	2023	Cui et al. (2023)
Ursolic acid	Nanoparticles	Chitosan	Oral cancer	2023	Karthik et al. (2023)
*Plectranthus amboinicus*, *Phyllanthus niruri*, and *Euphorbia hirta*	Metal NPs	Titanium dioxide	Oral cancer	2021	Maheswari et al. (2021)
Curcumin	Polymer	Polymer-stabilized nano-curcumin	Oral cancer	2022	Essawy et al. (2022)
Curcumin and *Dorema aucheri*	Liposome	DPPC and cholesterol	Oral cancer	2023	Azizi et al. (2023)

#### 4.1.1 Brain cancer

Waqar et al. co-loaded *Moringa oleifera* extract and Epilim into magnetite nanoparticles (MNPs) for magnetic targeting. The superparamagnetic MNPs (9 nm–23 nm) inhibited U87 cells at 10 mM, but the study did not establish brain exposure or survival benefit (Waqar et al., 2024). By contrast, Cui et al. evaluated a biomimetic exosome-based nanoplatform (CpG-EXO/TGM) and reported 2.74-fold greater transport across a blood–brain barrier (BBB) model than control nanoparticles and a median survival of 29 versus 15 days *in vivo* (Cui et al., 2023). Thus, only the latter provides an animal survival endpoint; neither study supports clinical efficacy.

#### 4.1.2 Oral cancer

Maria’s group prepared chitosan nanoparticles loaded with ursolic acid (UACNP) to enhance bioavailability and antioxidant activity for oral cancer chemoprevention. UACNP significantly suppressed DMBA-induced tumor formation by 67%, attenuated oxidative stress, and restored detoxification enzyme function, demonstrating efficacy in both preventive and therapeutic settings (Karthik et al., 2023). Maheswari’s study synthesized TiO_2_ nanoparticles modified with three different medicinal herbs (*Plectranthus amboinicus*, *Phyllanthus niruri* and *Euphorbia hirta*) via the hydrothermal method. The TiO_2_ nanoparticles modified with *P. amboinicus* and *P. niruri* showed the strongest anticancer activity, with 97% cell death against KB oral cancer cells at a concentration of 50 μg/mL. Further analysis by flow cytometry confirmed p53 upregulation (59.18 fluorescence intensity) while maintaining minimal cytotoxicity toward normal L929 cells (78% viability at 50 μg/mL) (Maheswari et al., 2021). Essawy et al. developed PVP-stabilized nanocurcumin via solvent–antisolvent precipitation, yielding 121-nm particles with an IC_50_ value of 60.8 μg/mL against oral squamous cell carcinoma cells (SCC4)—significantly lower than that of native curcumin (212.4 μg/mL). The nanocurcumin induced 85.5% late apoptosis at 24 h, inhibited cell migration by 25%, and showed high selectivity (index = 4.5) over normal fibroblasts, aided by enhanced luminescence-based cellular uptake (Essawy et al., 2022). The nanoliposomal delivery system co-encapsulating *Dorema aucheri* extract and curcumin markedly improved anticancer activity against oral cancer cells (OCC-02), reducing the IC_50_ to 43 μg/mL at 72 h, inducing 95% apoptosis, and inhibiting *EGFR* expression by 81%. This approach improved bioavailability and sustained release, demonstrating strong therapeutic potential (Azizi et al., 2023).

Taken together, these studies indicate carrier-dependent gains in cellular uptake and apoptosis, but the evidence is not equivalent across platforms. The glioblastoma study included an *in vivo* survival endpoint, whereas most oral cancer studies relied on cell viability or chemically induced tumor models. Differences in payload, dose, exposure time, and model, therefore, preclude direct potency rankings, and none of the studies establish clinical benefit.

### 4.2 Respiratory system neoplasms

Evidence for respiratory system cancers is concentrated in lung cancer models (Table 2). The studies encompass green-synthesized metals, lipid carriers, polymeric composites, protein carriers, and compound-based nanosystems, with reported mechanisms including reactive oxygen species (ROS) generation, apoptosis, cell-cycle arrest, and immune modulation (Zhang et al., 2024; Xu et al., 2025). However, most datasets use a limited set of cell lines and rarely provide formulation-matched pharmacokinetic or biodistribution comparisons.

**TABLE 2:** TCM nano-formulations for the treatment of respiratory system neoplasms.

TCM/Active components of TCM	Types of NPs	Materials of NPs	Types of cancer treated	Years	References
*Curcuma kwangsiensis* leaf aqueous extract	Metal NPs	Silver nitrate	Adenocarcinoma of the lung	2023	Liu et al. (2023)
*Enicostema axillare* leaf extract	Metal NPs	Selenium	Non-small-cell lung cancer	2021	Perumal et al. (2021)
Silymarin	Lipid-based NPs	Lipid	Non-small-cell lung cancer	2021	Sezer (2021)
*Aloe perryi* extract	Lipid-based NPs	Lipid and chitosan	Non-small-cell lung cancer	2023	Aldayel et al. (2023)
Ginger rhizome extract	Nanocomposites	Zirconium oxide-doped zinc oxide and reduced graphene oxide	Non-small-cell lung cancer	2023	Ahamed et al. (2023)
*Cucumis prophetarum* extract	Protein-based NPs	Bovine serum albumin	Non-small-cell lung cancer	2021	Hemlata et al. (2021)

#### 4.2.1 Lung cancer

##### 4.2.1.1 Metal nanoparticles

Silver nanoparticles (AgNPs) synthesized with an aqueous *Curcuma kwangsiensis* leaf extract had mean diameters of 15 nm–21 nm and produced dose-dependent cytotoxicity in HLC-1, LC-2/ad, and PC-14 lung cancer cells (IC_50_ values of 249, 187, and 152 μg/mL, respectively) while sparing HUVEC controls (Liu et al., 2023). In a separate study, Perumal et al. used *Enicostema axillare* leaf extract to synthesize selenium nanoparticles (SeNPs; 56 nm–98 nm). The particles inhibited A549 cell growth in a dose-dependent manner; zebrafish testing yielded an LC50 of 258.72 ppm and showed reduced Na+/K + -ATPase activity at higher exposure, supporting preliminary biocompatibility but not an apoptosis-specific mechanism (Perumal et al., 2021).

##### 4.2.1.2 Lipid-based nanoparticles

Sezer et al. constructed silymarin-loaded SLNs that measured 138.4 nm and −15.8 mV, achieving 82.3% encapsulation and a 91% sustained release over 72 h (Sezer, 2021). These SLNs induced G1 phase arrest and suppressed the Akt/CREB/MAPK axis in A549 cells, reducing the tumor volume by ≥ 50% through downregulation of *FOXM1* and *POLE2* (Sezer, 2021). Aldayel et al. engineered SLNs that were coated with chitosan and incorporated with *Aloe perryi* extract (C-ALP-SLNs) (Aldayel et al., 2023). The NPs (173.6 nm, 13.6 mV) displayed pH-dependent release kinetics (78% release at acidic pH 5) and potent cytotoxicity against A549 cells (IC_50_ = 11.42 μg/mL) by inhibiting *CXCL2* and myeloperoxidase expression to reduce neutrophil infiltration (Aldayel et al., 2023).

##### 4.2.1.3 Polymeric nanocomposites

Ahamed et al. synthesized ZnO/ZrO_2_/rGO nanocomposites (NCs) using ginger rhizome extract (Ahamed et al., 2023). The NCs (92.5 nm, −7.68 mV) showed dose-dependent cytotoxicity in A549 cells (IC_50_ = 25 μg/mL) by generating ROS and depleting glutathione (GSH) and activating the ROS/ASK1/JNK pathway and mitochondrial caspase cascade.

##### 4.2.1.4 Protein-based nanoparticles

Hemlata et al. prepared bovine serum albumin (BSA) nanospheres encapsulating *Cucumis prophetarum* fruit extract (Cp-BSA NPs; 188.5 nm; 82.3% encapsulation). In A549, HepG2, and MCF-7 cells, the formulation induced ROS-dependent mitochondrial apoptosis and remained less toxic to HEK293 cells; however, the evidence was restricted to *in vitro* models and did not establish lung-specific pharmacokinetics or tumor delivery (Hemlata et al., 2021).

##### 4.2.1.5 Natural compound-based nanosystems

Xu et al. described berberine hydrochloride nanoparticles (BHCNPs; 85.2 nm; −18.3 mV) that inhibited HMG-CoA reductase and mevalonate-pathway signaling in A549 and H1299 cells, with an IC_50_ of 12.4 μM and disruption of Ras/Rho protein prenylation (Xu et al., 2025). Because the cited source is a broader review of the use of berberine in lung cancer rather than a formulation-specific primary report, these quantitative claims should be interpreted cautiously.

Across the lung-cancer studies, nanoformulations improved the formulation properties or antitumor readouts in preclinical models, but efficacy estimates cannot be directly compared because the payloads, dose units, exposure times, and endpoints differed. Green-synthesized metal systems often showed cytotoxicity across several cell lines, whereas lipid and protein carriers provided clearer encapsulation or release data; few studies combined these endpoints with *in vivo* pharmacokinetics, organ distribution, and carrier-only controls. The evidence, therefore, supports further comparative preclinical evaluation rather than claims of established tumor targeting or reduced systemic toxicity.

### 4.3 Digestive system cancers

Studies of digestive system cancers include esophageal, gastric, colorectal, liver, bile duct, and pancreatic models (Table 3). The evidence is heterogeneous: some reports evaluate green-synthesized particles or carrier-free assemblies, whereas others test biodegradable polymers or complex co-delivery systems. This diversity broadens mechanistic coverage but limits direct comparison of potency and translational readiness.

**TABLE 3:** TCM nano-formulations for the treatment of digestive system cancer.

TCM/Active components of TCM	Types of NPs	Materials of NPs	Types of cancer treated	Years	References
*Cirsium japonicum* extract	Metal NPs	Gold	Gastric cancer	2022	Mi et al. (2022)
*Teucrium polium* extract	Metal oxide nanocomposites	Zinc oxide	Gastric cancer	2023	Bozgeyik et al. (2023)
*Ardisia gigantifolia*	Metal NPs	Silver	Gastric cancer	2023	Le et al. (2023)
*Mentha spicata* extract	Metal NPs	Iron	Colorectal cancer	2024	Nejad et al. (2024)
Rosemary and ginseng extracts	Bimetallic NPs	Palladium and platinum	Colorectal cancer	2024	Alinaghi et al. (2024)
Dihydroartemisinin	Metal NPs	Metal–organic frameworks	Colorectal cancer	2024	Yang L. et al. (2024)
*Bacopa monnieri*	Metal NPs	Gold	Colorectal cancer	2024	Meher et al. (2024)
Ginger extract	Metal oxide nanocomposites	Platinic oxide	Colorectal cancer	2024	Cherik et al. (2024)
Garlic derivatives	Nanocomposites	Garlic derivatives	Colorectal cancer	2024	Xu et al. (2024)
Ursolic acid and lentinan	Self-assembled NPs	Ursolic acid and lentinan	Colorectal cancer	2022	Mao et al. (2022)
Genistein	Metal NPs	Zinc ferrite	Liver cancer	2023	Otuechere et al. (2023)
*Morinda officinalis* polysaccharides	Inorganic NPs	Selenium	Liver cancer	2023	Yao et al. (2023)
*Ephedra aphylla* extract	Inorganic NPs	Selenium and zinc	Liver cancer	2021	El-Zayat et al. (2021)
Rosmarinic acid	Self-assembled NPs	Iron	Liver cancer	2024	Chen Y. et al. (2024)
*Artemisia carvifolia* Buch. extract	Inorganic NPs	Silver	Liver cancer	2023	Javid and Dilshad (2023)
Boswellic acid, curcumin, and naringenin	Organic NPs	PLGA	Liver cancer	2023	Elnawasany et al. (2023)
*Ficus carica* leaf extract	Organic NPs	PLGA	Bile duct cancer	2025	Aziz et al. (2025)
*Myrica esculenta* plant extract	Bionic NPs	Albumin	Pancreatic cancer	2024	Shukla et al. (2024)
Pectin	Nanocomposites	Tannin	Pancreatic cancer	2020	Chauhan et al. (2020)

#### 4.3.1 Esophageal cancer

Zhao et al. evaluated the efficacy of free luteolin in paclitaxel-resistant esophageal cancer models, where 40 μM free luteolin reduced sphere formation and SOX2 abundance. *In vivo*, luteolin reduced tumor formation from 20% in controls to 0%; however, this study assessed free luteolin rather than its nanoformulation and, therefore, provides a mechanistic context, not direct evidence of nanocarrier benefit (Zhao et al., 2021).

#### 4.3.2 Gastric cancer

Mi’s work developed gold nanoparticles using *Cirsium japonicum* extract (CJ-AuNPs) via green synthesis, which exhibited significant anticancer effects on AGS gastric cancer cells by triggering ferroptosis. The optimized CJ-AuNPs reduced tumor growth in xenograft mice by 31.59%, 47.45%, and 62.49% at doses of 2.5, 5, and 10 mg/kg, respectively. Mechanistic investigations revealed that CJ-AuNPs promoted iron-dependent necrocytosis through ROS accumulation, elevated malondialdehyde (MDA) levels indicative of lipid peroxidation, mitochondrial dysfunction, downregulation of glutathione peroxidase-4 (GPX4), and concomitant upregulation of heme oxygenase-1 (HO-1). Furthermore, the nanoparticles demonstrated favorable biocompatibility, with hemolysis rates below 10% at 150 μg/mL and no observable systemic toxicity in living organisms (Mi et al., 2022). Bozgeyik et al. developed zinc oxide nanoparticles (TP-ZnO NPs) using *Teucrium polium* extract, which demonstrated significant anticancer effects on HGC-27 gastric cancer cells. These nanoparticles exhibited strong cytotoxicity (IC_50_ = 10.88 μg/mL), which is considerably less than that of cisplatin (33.28 μg/mL). At a concentration of 15 μg/mL, TP-ZnO-NPs induced robust apoptosis, with a late apoptotic cell ratio reaching 71.1%. They also exhibited dose-dependent inhibition of both cell migration and colony formation. Mechanistic analysis revealed that TP-ZnO-NPs significantly upregulated the expressions of pro-apoptotic genes, including *BAX, CASP9*, and *PTEN*, while concurrently downregulating the expression of the anti-apoptotic gene *BCL-2*. Furthermore, these NPs were observed to modulate epithelial–mesenchymal transition by upregulating E-cadherin expression and downregulating vimentin expression. These findings support further preclinical evaluation of TP-ZnO NPs in gastric cancer (Bozgeyik et al., 2023). Le’s research demonstrated the environmentally sustainable synthesis of AgNPs utilizing *Ardisia gigantifolia* leaf extract (Arg-AgNPs), which showed potent anticancer activity against AGS and MKN45 cell lines. The synthesized Arg-AgNPs exhibited a uniform size distribution of 6 nm and demonstrated dose-dependent cytotoxicity against gastric cancer cells, with IC_50_ values decreasing from 1.37 to 0.65 μg/mL (AGS cells) and from 1.03 to 0.96 μg/mL (MKN45 cells) over 24 h–48 h treatment periods. Functionally, Arg-AgNPs significantly arrested the cell cycle at the G0/G1 phase, inhibited cell migration, enhanced cellular senescence by 4.1–7.6-fold, and elevated ROS production by 2–15 times, indicating a potent antiproliferative mechanism mediated through oxidative stress (Le et al., 2023).

#### 4.3.3 Colorectal cancer

Nejad’s study developed iron nanoparticles (FeNPs) using *Mentha spicata* extract via green synthesis, which exhibited significant anticancer effects against LS 174T colon cancer cells. The FeNPs exhibited cytotoxicity that varied with dose and time as the IC_50_ values decreased from 2,740 μg/mL at 24 h to 574.5 μg/mL at 72 h under 15 MV radiation. Additionally, the nanoparticles triggered apoptosis by elevating BAX and downregulating Bcl-2 expressions, and they enhanced radiotherapeutic efficacy (Nejad et al., 2024). Bimetallic Pd/Pt nanoparticles were synthesized using rosemary and ginseng extracts, demonstrating potent anticancer activity against colorectal cancer lines SW480 and LS180. The IC_50_ values for the optimized NPs were 436.3 μg/mL against SW480 cells and 790 μg/mL against LS180 cells after a 24-h treatment period. They also modulated autophagy-related genes (*BECN1,* *MAP1LC3B,* and *SQSTM1*) in a dose- and time-dependent manner, indicating dual apoptotic and autophagic cell death mechanisms (Alinaghi et al., 2024). Yang’s study created a nano-herb system (PMDC NPs) that responds to both pH and GSH for targeted delivery of dihydroartemisinin (DHA) and carbon monoxide-releasing molecule (CORM-401) for treating colorectal cancer. The NPs exhibited high drug-loading efficiency and significantly induced immunogenic cell death, with IC_50_ values below 50 μg/mL in CT26 and RKO cells. *In vivo*, these NPs potently suppressed tumor growth by over 80%, enhanced T-cell infiltration accompanied by 30% mature dendritic cells in distant tumors, and prevented recurrence through ROS-/CO-mediated mechanisms, demonstrating great potential for combined chemo–gas-immunotherapy against colorectal cancer (Yang C. et al., 2024). Meher et al. reported Bm-GNPs that exhibited potent antiproliferative activity against HT-29 cells, inducing G_2_/M phase arrest in 35% of cells (Meher et al., 2024). They triggered caspase-independent autophagic cell death via tubulin binding and secondary structure disruption without altering tertiary conformation. Cherik et al. synthesized oxaliplatin nanoparticles (OxPt NPs) using ginger extract, obtaining particle sizes of 70 nm–114.5 nm and a zeta potential of 53 mV. The NPs showed lower IC_50_ values in HCT-116 cells than free oxaliplatin or ginger extract. Apoptosis accounted for 87.9% of treated cells; however, this *in vitro* endpoint does not establish tumor-targeting specificity (Cherik et al., 2024). Xu et al. developed oral garlic-derived nanoparticles (GNPs), which demonstrated a remarkable capacity to amplify and activate intestinal IFNγ-expressing γδ T cells, increasing the proportion among CD3^+^ T cells by approximately 2.5-fold and enhancing IFNγ production. Oral GNPs promoted the migration of these activated γδ T cells to distant tumors, significantly inhibiting tumor growth in multiple models (such as CT26 colon carcinoma) and improving survival, with nearly 80% tumor suppression when combined with anti-PD-L1 therapy (Xu et al., 2024). Mao et al. developed a self-assembled nanodrug named LNT-UA by combining lentinan with ursolic acid, both derived from TCM, through a nanoprecipitation method without requiring additional carriers. The nanodrug demonstrates a high UA loading capacity of 54.6% and excellent stability, with a uniform particle size of approximately 68.8 nm. It induces immunogenic cell death, increases the number of mature dendritic cells to 35.3%, shifts macrophages to the M1 phenotype with a higher M1/M2 ratio, and nearly doubles CD8^+^ T-cell infiltration, thus effectively transforming the immunosuppressive tumor environment in colorectal cancer (Mao et al., 2022).

#### 4.3.4 Liver cancer

Otuechere et al. fabricated genistein-functionalized zinc ferrite nanoparticles (GENPs) through eco-friendly synthesis mediated by *Pterocarpus mildbraedii* leaf extract. The GENP exhibited significant antiproliferative activity against HepG2 cells *in vitro*, reducing the cell viability to 46.9% ± 0.6 at 250 μg/mL, while demonstrating low toxicity in normal THLE-3 cells (84.9% ± 0.6 viability at the same concentration). *In vivo* results in a chemically induced (diethylnitrosamine and 2-N-acetyl-2-amino-fluorene) rat model confirmed that GENP effectively restored hepatic and renal function markers, and molecular docking revealed genistein’s strong binding affinity to MMP-9 (−19.0 ± 0.6 kcal/mol), comparable to that of the standard inhibitor marimastat (Otuechere et al., 2023). Yao et al. synthesized and stabilized SeNPs using *Morinda officinalis* polysaccharides (Se-MOP), resulting in highly stable and uniformly dispersed particles. Se-MOP nanoparticles exhibited potent anticancer activity, particularly against HepG2 cells (IC_50_ = 1 μg/mL), and induced G0/G1 phase arrest. They also significantly enhanced immune function by promoting lymphocyte proliferation and upregulating the expressions of key cytokines such as IL-2 (Yao et al., 2023). EL-Zayat et al. synthesized selenium–zinc composite NPs using *Ephedra aphylla* extract for application in antitumor therapy. The SeNPs demonstrated potent cytotoxicity against a variety of cancer types, with IC_50_ values recorded as low as 7.56 ± 0.6 μg/mL for HepG2 cells, 9.23 ± 0.8 μg/mL for HeLa cells, and 10.02 ± 0.9 μg/mL for HCT-116 cells (El-Zayat et al., 2021). Chen et al. developed a self-assembled Fe–rosmarinic acid nanocomposite (Fe-RA) that exhibits peroxidase-like activity and depletes GSH in the tumor microenvironment. Fe-RA demonstrated efficient catalytic performance, with a Vmax of 0.5166 μM s^−1^ for TMB oxidation and 6.147 μM s^−1^ for GSH consumption, while inducing significant ferroptosis in H22 cells. *In vivo*, this treatment markedly inhibited tumor growth, reducing tumor volume and weight, and downregulated the expressions of key ferroptosis-related proteins including GPX4 and SLC3A2 (Chen Y. et al., 2024). Javid and Dilshad synthesized AgNPs through a green synthesis using *Artemisia carvifolia* Buch extract. Biosynthesized AgNPs exhibited potent anticancer effects on HepG2, with a notably low IC_50_ value (2.57 µM). Further analysis revealed a marked reduction in the oncogene *RAP2A* coupled with elevated levels of proapoptotic markers such as Bax and caspases, demonstrating activation of both intrinsic and extrinsic apoptotic pathways. These promising findings were supported by protein-level evidence, indicating AgNPs as a potent treatment strategy for liver cancer (Javid and Dilshad, 2023). Elnawasany et al. developed PLGA nanoparticles co-encapsulating boswellic acids, curcumin, and naringenin via nanoprecipitation, significantly enhancing the antiproliferative effects on HepG2. The nanoparticles demonstrated significantly lower IC_50_ values than the free compounds: boswellic acid (5.58 ± 0.27 vs. 22.45 ± 1.13 μg/mL), curcumin (2.51 ± 0.11 vs. 5.57 ± 0.94 μg/mL), and naringenin (5.21 ± 0.18 vs. 11.37 ± 1.45 μg/mL) at 48 h. The improvement was attributed to enhanced cellular delivery and controlled release from the nanoformulations (Elnawasany et al., 2023). Across these studies, the apparent potency varied by orders of magnitude, but direct ranking is inappropriate because formulations, exposure units, cell lines, and the endpoints differed. *In vivo* pharmacokinetics and long-term organ retention were reported inconsistently, particularly for metal-based carriers.

#### 4.3.5 Bile duct cancer

Aziz et al. formulated PLGA nanoparticles infused with *Ficus carica* leaf extract, enhancing their anticancer efficacy on extrahepatic cholangiocarcinoma TFK-1 cells. The optimized NPs exhibited a uniform size of 162 ± 0.7 nm with an encapsulation efficiency of 56% ± 2.3%. Importantly, the nanoformulation demonstrated significantly enhanced therapeutic effects, showing a cytotoxicity IC_50_ value of 0.6 mg/mL after 48 h compared to 1.0 mg/mL for the free extract, and a phototoxicity IC_50_ value of 42 μg/mL after 6 h versus 67 μg/mL for the free extract. Furthermore, the FCe-loaded NPs produced more pronounced G0/G1 cell-cycle arrest and promoted superior cellular uptake and nuclear localization compared to the free extract (Aziz et al., 2025).

#### 4.3.6 Pancreatic cancer

Shukla et al. developed and evaluated NPs synthesized from *Myrica esculenta* bark extract and bovine serum albumin (ME-BSANPs) for cancer therapy. These NPs demonstrated significant cytotoxicity in pancreatic cancer cells (MIA PaCa-2), with a cell growth inhibition rate of 40% ± 3.7% after 48 h of treatment. Moreover, they exhibited minimal toxicity toward normal HEK-293 cells, indicating high selectivity. Molecular docking studies revealed strong binding affinity between active constituents of *M. esculenta* (such as tannic acid and myricetin) and BSA, with binding energies as low as −10.163 kcal/mol, suggesting a potential mechanism for enhanced drug targeting and apoptosis induction in cancer cells (Shukla et al., 2024). Chauhan et al. developed stable nanocomplexes (MPT-NCs) from modified pectin and tannic acid for targeted drug delivery in pancreatic cancer. The MPT-NCs had an optimal particle size of 80 nm–154 nm, exhibited high drug encapsulation efficiency (>92%), and demonstrated dose- and time-dependent cellular uptake in pancreatic cancer cells (HPAF-II and PANC-1). Drug-loaded MPT-NCs significantly enhanced anticancer efficacy, with IC_50_ values for gemcitabine being reduced from 107.5 nM (free drug) to 70.74 nM in HPAF-II cells, and they also strongly inhibited colony formation compared to free drugs (Chauhan et al., 2020).

Overall, the evidence for digestive system cancers remains predominantly preclinical. Direct comparison is limited by differences in tumor models, exposure units, treatment schedules, and endpoints, and many reports lack free-compound, blank-carrier, or standard-of-care controls. Metal and metal-oxide systems often produce low *in vitro* IC_50_ values but raise unresolved questions about persistence and organ accumulation; biodegradable polymeric or self-assembled carriers provide clearer formulation advantages but still lack standardized pharmacokinetic and long-term safety data. Future studies should use common comparators, clinically relevant models, scalable methods, and quantitative biodistribution.

### 4.4 Reproductive system cancers

Research on TCM nanoformulations for reproductive system cancers remains at an early stage and is concentrated on breast cancer, with fewer studies on cervical and prostate cancer (Table 4). These formulations may address multidrug resistance, induce tumor cell apoptosis, and inhibit metastasis. However, reported improvements in targeting efficiency or efficacy require confirmation with formulation-matched controls and quantitative biodistribution data.

**TABLE 4:** TCM nano-formulations for the treatment of reproductive system cancer.

TCM/Active components of TCM	Types of NPs	Materials of NPs	Types of cancer treated	Years	References
*B. javanica*	Nanovesicle	Exosome-mimicking nanovesicles obtained from *B. javanica*	Triple-negative breast cancer	2024	Yan et al. (2024)
Celastrol and erianin	Self-assembled NPs	Celastrol and erianin	Breast cancer	2024	Tian et al. (2024)
*Garcinia mangostana* extract	Inorganic NPs	Silver	Breast cancer	2022	Alobaid et al. (2022)
Baicalein	Organic NPs	PEG-PLGA	Breast cancer	2023	Zheng et al. (2023)
Curcumin	Self-assembled NPs	Gelatin, oleic acid, and carboxymethyl chitosan	Breast cancer	2025	Sivasankaran et al. (2025)
Limonene	Organic NPs	Chitosan	Breast cancer	2021	Alipanah et al. (2021)
Diosgenin	Polymer	PLGA-DGN	Breast cancer	2022	Dey et al. (2022)
*Platycodon grandiflorum* and *Curcuma zedoaria*	Polymer	Lipid	Breast cancer	2024	Shi et al. (2024)
*Aloe*	Inorganic NPs	Silver	Breast cancer	2024	Sudhimon et al. (2024)
*Olea europaea* fruit extract	Inorganic NPs	Silver	Breast cancer	2023	Alharbi and Felimban (2023)
*Rheum emodi*	Metal oxide nanocomposites	Stannic oxide	Breast cancer	2021	Sharma et al. (2021)
*Dendrobium* polysaccharides	Self-assembled NPs	*Dendrobium* polysaccharides	Breast cancer	2025	Tao et al. (2025)
Osthole	Metal oxide nanocomposites	Silicon dioxide	Cervical cancer	2024	Chen et al. (2024)
*Annona muricata* L. and *Caesalpinia sappan* L.	Self-assembled NPs	*Annona muricata* L. and *Caesalpinia sappan* L.	Cervical cancer	2023	Astirin et al. (2023)
Curcumin	Self-assembled NPs	Glucan	Prostatic cancer	2021	Bevacqua et al. (2021)
Deer antler extract	Self-assembled NPs	Deer antler extract	Prostatic cancer	2024	Nazarbek et al. (2024)

#### 4.4.1 Breast cancer

Breast cancer represents a significant global health issue characterized by heterogeneous subtypes and high propensity for metastasis and drug resistance. The complexity of breast cancer pathogenesis, especially multidrug resistance (MDR), challenges conventional therapies. Nano-formulated TCMs offer innovative solutions through enhanced targeting, enabling co-delivery and modulating key signaling pathways, thus providing new avenues for overcoming therapeutic resistance and improving treatment outcomes (Yan et al., 2024; Tian et al., 2024; Alobaid et al., 2022).

##### 4.4.1.1 Polymeric nanoparticles

Polymeric nanoparticles, primarily composed of biodegradable substances, facilitate controlled drug release and site-specific delivery. Zheng et al. loaded baicalein into mPEG-PLGA NPs (PMs-Ba) (Zheng et al., 2023). In a 4T1 breast cancer model, these NPs targeted TGF-β signaling to inhibit the activation of cancer-associated fibroblasts (CAFs), alter tumor immune microenvironments, and cooperatively increase the antitumor efficacy of doxorubicin (DOX) nanoparticles (PMs-ADM). Sivasankaran et al. engineered self-assembled NPs composed of gelatin, oleic acid, and carboxymethyl chitosan (GOCNPs) to transport curcumin (Sivasankaran et al., 2025). The GOCNPs exhibited high drug loading, pH-/enzyme-responsive release, and significant cytotoxicity on MCF-7 cells (IC_50_ = 6.88 μg/mL), showcasing great potential as an oral delivery system. Tian et al. engineered NPs that self-assemble from celastrol and erianin (CEN) without crosslinkers (Tian et al., 2024). In 4T1 breast cancer cells, the CEN NPs induced G2/M cell-cycle arrest and blocked invasion, pinpointing Annexin A2 as a direct molecular target of celastrol. Alipanah et al. synthesized chitosan NPs loaded with limonene via ionic gelation (Alipanah et al., 2021). These NPs were cytotoxic to MDA-MB-468 cells (IC_50_ = 23.65 μg/mL) and induced apoptosis through caspase activation. Dey et al. synthesized poly(lactic-co-glycolic acid) (PLGA)-encapsulated diosgenin nanoparticles (PLGA-DGN NPs) employing an adapted nanoprecipitation technique (Dey et al., 2022). The NPs increased cellular absorption and toxicity in MCF-7 cells and showed favorable pharmacokinetics and antiangiogenic effects *in vivo*. Sezer et al. fabricated silymarin-loaded SLNs via hot homogenization (Sezer, 2021). The SLNs led to higher levels of cytotoxicity and apoptosis induction in MCF-7 cells than silymarin alone. Yan et al. extracted nanovesicles resembling exosomes from *Brucea javanica*, referred to as BF-Exos (Yan et al., 2024). These nanovesicles delivered active miRNAs to 4T1 cells, suppressing tumor growth and spread by modulating the PI3K/Akt/mTOR pathway and boosting immune responses. Shi et al. constructed an herb–nanoparticle hybrid system (HNS) by attaching *Platycodon grandiflorum* and *Curcuma zedoaria* extract to lipid–polymer NPs containing silibinin (Shi et al., 2024). The HNS facilitated better distribution in tumor tissues, alleviating lung metastasis in a 4T1 model.

##### 4.4.1.2 Metallic nanoparticles

Metallic nanoparticles, particularly AgNPs synthesized via green methods, are investigated for their unique anticancer properties. Sudhimon et al. biosynthesized AgNPs using *Aloe barbadensis* Miller extract (Sudhimon et al., 2024). The AgNPs exhibited concentration-dependent cytotoxicity against breast cancer cells by triggering ROS-mediated apoptosis and halting the cell cycle. Alharbi et al. optimized AgNP synthesis with *Olea europaea* fruit extract (Alharbi and Felimban, 2023). The AgNPs showed dose-dependent cytotoxicity against MCF-7 and T47D cells and induced apoptosis and DNA fragmentation. Alobaid et al. biosynthesized AgNPs using *Garcinia mangostana* (mangosteen) peel extract (Alobaid et al., 2022). The cytotoxicity of spherical AgNPs was dose-dependent, with an IC_50_ of 50 μg/mL against MCF-7, resulting in apoptosis and increased ROS levels. Sharma et al. adopted an eco-friendly approach to generate tin oxide nanoparticles (SnO_2_ NPs) from *Rheum emodi* root extract (Sharma et al., 2021). SnO_2_ NPs demonstrated harmful and apoptosis-inducing effects on MDA-MB-231 cells.

##### 4.4.1.3 Other nanoformulations

Tao et al. developed folic acid-conjugated *Dendrobium officinale* polysaccharide NPs for DOX delivery (FA@Dox NPs) (Tao et al., 2025). The NPs displayed pH-responsive release, enhanced cytotoxicity against FR-rich breast cancer cells (4T1, CT26), and promoted immunogenic cell death (ICD), successfully suppressing tumor growth and spread *in vivo*.

#### 4.4.2 Cervical cancer

Chen et al. developed a tumor-targeted nano-herbal delivery system, HMOA NPs, specifically engineered to deliver both osthole and L-arginine concurrently, enabling synergistic chemotherapy and gas therapy against cervical cancer. The formulated NPs exhibited favorable physicochemical properties, including a high encapsulation efficiency of 75.70% for osthole and 64.40% for L-arginine, and demonstrated glutathione-triggered release profiles. *In vitro*, HMOA NPs reduced cervical cancer cell viability to 36% in HeLa and 30% in SiHa cells, while normal LO2 cells retained over 90% viability, indicating low toxicity. In a murine model, this targeted nanoplatform provided 90.80% tumor inhibition, significantly decreased metastasis by modulating epithelial–mesenchymal transition markers, and showed outstanding biosafety in systemic assessments (Chen L. et al., 2024). Astirin et al. investigated the cytotoxicity of soursop leaf (*Annona muricata* L.) and sappan wood (*Caesalpinia sappan* L.) extracts against HeLa cells. They compared fractions, isolates, and chitosan-based NPs. The MTT assay demonstrated that the fraction and isolate forms possessed superior cytotoxic activity, yielding IC_50_ of 7.13 μg/mL and 8.17 μg/mL for soursop leaf and 13.07 μg/mL and 6.9 μg/mL for sappan wood, respectively. In contrast, the IC_50_ values for the NP formulations were significantly elevated at 88.98 μg/mL and 82.53 μg/mL, respectively, indicating reduced potency in the single-dose treatment. Flow cytometric analysis confirmed the induction of both apoptosis and necrosis across all sample types. Notably, when combined at 1/10 IC_50_, the NPs demonstrated a synergistic effect, as evidenced by a combination index of 0.54 (Astirin et al., 2023).

#### 4.4.3 Prostate cancer

Self-assembling dextran-curcumin nanoparticles (DEX/CUR) increased the apparent cytotoxicity of curcumin in PC-3 cells, reducing the IC_50_ from 27.24 to 12.21 μM and increasing ROS generation from 67% to 134% at 10 μM curcumin-equivalent exposure. The system also enabled acidic-pH release of doxorubicin and altered the epithelial–mesenchymal transition markers, but these findings were obtained *in vitro* and do not establish pharmacokinetics, biodistribution, or tumor targeting (Bevacqua et al., 2021). Guldan’s work demonstrated that deer antler extract contains NPs (150 nm–250 nm, nearly −1.236 mV) exhibiting co-nanozyme activities, including catalase and phosphatase-like functions, particularly at pH 13. A significant reduction in prostate cancer cell (DU 145 and PC-3) viability was induced by the antler extract. Moreover, a potent synergy with the MET kinase inhibitor crizotinib was also observed, which significantly augmented the anticancer effect. Network pharmacology and molecular docking suggested that prostaglandin E in the extract binds strongly to MET kinase (binding energy: 12.162 kcal/mol), indicating a potential mechanism for its targeted anticancer activity (Nazarbek et al., 2024).

In summary, current research studies on reproductive system cancers are increasingly emphasizing the design of natural product-derived nanocarriers, with particular focus on breast, cervical, and prostate malignancies. These drug delivery systems show improved therapeutic effectiveness and targeting precision, effectively inhibiting metastasis in cell models. Nonetheless, there are still obstacles, including occasionally reduced potency compared to pure extracts, a lack of extensive *in vivo* data, and the necessity to extend these strategies to other reproductive cancers that have been less researched. Upcoming research should focus on applying these results to clinical trials, refining combination therapies to tackle decreased effectiveness, and delving deeper into molecular mechanisms for broader use.

### 4.5 Other cancers

#### 4.5.1 Melanoma

Alipanah et al. developed chitosan-based NPs encapsulating *Citrus sinensis* and *Citrus limon* essential oils via ionic gelation (Alipanah et al., 2021). With sizes of 156 ± 8 nm and 181 ± 4 nm, the spherical NPs showed strong cytotoxicity toward A-375 melanoma cells, with IC_50_ values of 0.03 and 0.124 μg/mL, respectively. The formulation showed high biocompatibility with normal cells, indicating selective targeting and apoptosis induction via ROS-mediated pathways (Alipanah et al., 2021). Ibrahim et al. formulated thymoquinone (TQ)-encapsulated PLGA NPs using a solvent evaporation technique (Ibrahim et al., 2020). The optimized TQ-PLGA NPs exhibited a particle size of 147.2 nm, a zeta potential of +22.1 mV, and an encapsulation efficiency of 96.8%. *In vitro* release profiles demonstrated an initial burst release of 45.6% within the first 3 h, followed by sustained drug release extending over 7 days. Cellular uptake in A375 cells was time-dependent, and significant cytotoxicity was observed within 24 h, which is attributed to rapid TQ release and ROS generation (Ibrahim et al., 2020). Li et al. engineered celastrol (CEL)-conjugated mPEG-PLL NPs using a self-assembly method prompted by *in situ* chemical conjugation (Li et al., 2020). CEL-NPs had a hydrodynamic radius of 65 ± 6.4 nm and exhibited sustained drug release (∼60% over 48 h). In B16F10 tumor-bearing mice, CEL-NPs significantly suppressed tumor growth by modulating apoptotic proteins (Bax and Bcl-2) and reducing systemic toxicity compared to free CEL (Li et al., 2020). Wu’s group biosynthesized AuNPs using Siberian ginseng extract (SG-GNPs) via a green synthesis approach (Wu et al., 2019). The SG-GNPs were spherical, crystalline, and sized ∼200 nm. They triggered apoptosis in B16 melanoma cells by boosting ROS production, reducing mitochondrial membrane potential, and upregulating caspase-3 and caspase-9 expressions (Wu et al., 2019).

#### 4.5.2 Bladder cancer

Using *Panax zingiberensis* leaf extract, Liu et al. produced AgNPs (Liu and Sun, 2025). The AgNPs exhibited a spherical morphology and a mean hydrodynamic diameter of 43.19 nm, with a characteristic surface plasmon resonance absorption peak observed at 460 nm. They exhibited dose-dependent cytotoxicity toward HT-1376, TCCSUP, SCaBER, and UM-UC-3 bladder cancer cells, with IC_50_ values from 101 to 274 μg/mL, and demonstrated antioxidant activity in a DPPH assay (IC_50_ = 37 μg/mL) (Liu and Sun, 2025).

### 4.6 Bone tissue engineering and antibacterial applications

Bibi et al. fabricated mesoporous bioactive glass nanoparticles (MBGNs) containing *Peganum harmala* extract (PH-MBGNs) using a modified Stöber method (Bibi et al., 2023). Characterized by a spherical morphology, the NPs measured 180 ± 15 nm and had a surface area of 606 m^2^/g. In simulated body fluid, PH-MBGNs formed a hydroxyapatite coating and inhibited the proliferation of *S. aureus* and *E. coli*, suggesting potential for bone regeneration and infection control in osteomyelitis (Bibi et al., 2023).

### 4.7 Broad-spectrum anticancer and photocatalytic applications

Anigol et al. synthesized AgNPs with adjustable pH using *Capparis moonii* fruit extract under microwave irradiation (Anigol et al., 2023). The NPs ranged from 10 to 20 nm and showed pH-dependent anticancer activity against MCF-7 (IC_50_ = 12.06 μg/mL), A549 (IC_50_ = 24.87 μg/mL), and other cancer lines. Additionally, the NPs effectively catalyzed the breakdown of Rose Bengal and methylene blue dyes when exposed to sunlight (Anigol et al., 2023).

The studies grouped in Table 5 demonstrate the breadth rather than a uniform level of evidence. Melanoma formulations include controlled-release and animal data, whereas the bladder cancer and broad-spectrum AgNP studies are largely cell-based; the bone application addresses regeneration and infection control rather than direct antitumor efficacy. These distinct endpoints should not be pooled as evidence of generalized cancer targeting. Material persistence, carrier controls, long-term safety, and disease-specific *in vivo* validation remain key gaps.

**TABLE 5:** TCM nano-formulations for the treatment of other cancer.

TCM/Active components of TCM	Types of NPs	Materials of NPs	Types of cancer treated	Years	References
Thymoquinone	Organic NPs	PLGA	Melanoma	2020	Ibrahim et al. (2020)
Thunder God Vine	Organic NPs	PEG	Melanoma	2020	Li et al. (2020)
Ginseng	Metal NPs	Gold	Melanoma	2019	Wu et al. (2019)
*Panax zingiberensis* leaf extract	Metal NPs	Silver	Bladder cancer	2025	Liu and Sun (2025)
*Sophora flavescens* root	Inorganic NPs	Silicon	Bone cancer	2023	Bibi et al. (2023)
Lavender extract	Metal NPs	Silver	Cutaneous carcinoma	2023	Anigol et al. (2023)

To enable cross-platform comparison, Table 6 summarizes representative TCM compounds by nanocarrier, cancer type, mechanism of action, advantage, and limitation. The comparison shows that efficacy signals are often strong *in vitro* or in xenografts, whereas quantitative pharmacokinetics, organ distribution, manufacturability, and long-term safety are frequently missing.

**TABLE 6:** Comparative summary of representative TCM nanoformulations: mechanisms, advantages, and limitations.

TCM compound	Nanocarrier	Cancer type	Mechanism of action	Advantage	Limitation
Tanshinone IIA + glycyrrhizic acid (Cui et al., 2023)	Biomimetic exosome-based nanoplatform	Glioblastoma	BBB traversal with combined chemo-immunotherapy	2.74-fold higher transport than control NPs; median survival 29 vs. 15 days	Complex multicomponent platform; efficacy demonstrated in animal models
Ursolic acid (Karthik et al., 2023)	Chitosan nanoparticles	Oral cancer	Reduces oxidative stress and restores detoxification enzymes	Suppressed DMBA-induced tumor formation by 67%; biodegradable carrier	Hamster carcinogenesis model; systemic PK and organ distribution not reported
Curcumin (Essawy et al., 2022)	PVP-stabilized nanocurcumin	Oral squamous cell carcinoma	Enhanced uptake, apoptosis induction, and migration inhibition	IC_50_ 60.8 vs. 212.4 μg/mL for native curcumin; selectivity index 4.5	Primarily *in vitro* evidence; scale-up, exposure, and clearance remain undefined
Silymarin (Sezer, 2021)	Solid lipid nanoparticles	Non-small-cell lung cancer	G1 arrest; suppression of Akt/CREB/MAPK, FOXM1, and POLE2	82.3% encapsulation and sustained release; tumor volume reduced by at least 50%	Limited preclinical models; RES uptake and comparative PK were not quantified
Dihydroartemisinin + CORM-401 (Yang C. et al., 2024)	pH-/GSH-responsive nano-herb platform	Colorectal cancer	ROS-/CO-mediated immunogenic cell death and T-cell activation	More than 80% tumor suppression and prevention of recurrence *in vivo*	Complex co-delivery and manufacturing; human safety and PK are unknown
Lentinan + ursolic acid (Mao et al., 2022)	Carrier-free self-assembled nanodrug	Colorectal cancer	ICD, dendritic-cell maturation, M1 polarization, and CD8 T-cell recruitment	54.6% ursolic acid loading and broad tumor microenvironment remodeling	Stability, immunogenicity, repeat-dose exposure, and clinical reproducibility need study
*Brucea javanica* components (Yan et al., 2024)	Plant-derived exosome-like nanovesicles	Triple-negative breast cancer	Active miRNA delivery suppressing PI3K/Akt/mTOR with immune activation	Biomimetic carrier with antitumor and antimetastatic activity	Source heterogeneity, cargo definition, potency assays, and batch control are challenging
Baicalein (Zheng et al., 2023)	mPEG-PLGA micelles	Breast cancer	Inhibits TGF-β-driven CAF activation and improves doxorubicin activity	Combines stromal remodeling with chemotherapy	Combination design obscures individual contribution; human PK and biodistribution absent
Osthole + L-arginine (Chen L. et al., 2024)	GSH-responsive HMOA nanoparticles	Cervical cancer	Synergistic chemotherapy/gas therapy and EMT modulation	90.8% tumor inhibition; greater than 90% viability in normal LO2 cells	Murine evidence only; long-term material fate and gas-therapy safety need evaluation
Curcumin/dextran + doxorubicin (Bevacqua et al., 2021)	Self-assembled dextran–curcumin nanoparticles	Prostate cancer	ROS generation, acidic-pH drug release, and EMT suppression	Curcumin IC_50_ reduced from 27.24 to 12.21 μM; 90% DOX release at acidic pH	Predominantly *in vitro*; *in vivo* PK, organ distribution, and carrier contribution unresolved
Celastrol (Li et al., 2020)	mPEG-PLL self-assembled nanoparticles	Melanoma	Bax/Bcl-2-mediated apoptosis	Sustained release and lower systemic toxicity than free celastrol in mice	Narrow therapeutic index and polymer clearance require clinical-grade evaluation

## 5 Discussion

Nanoformulations can address important pharmacological limitations of TCM-derived constituents, such as inadequate solubility, swift systemic clearance, and poor tumor accumulation (Ai et al., 2024; Fu et al., 2025; Singh et al., 2024). Nanocarriers such as liposomes, polymeric nanoparticles, micelles, mesoporous silica, and metallic NPs facilitate efficient encapsulation, safeguarding, and transport of TCM compounds (Ai et al., 2024; Zielińska et al., 2020; Singh et al., 2024). By leveraging passive targeting, which is mediated by the EPR effect, along with active targeting via surface-bound ligands, these nanosystems boost therapeutic efficacy. These mechanisms may increase tumor exposure, although improved delivery cannot be assumed without quantitative biodistribution data (Yang L. et al., 2024; Zielińska et al., 2020). However, each platform entails distinct trade-offs. Liposomes offer high biocompatibility and scalable manufacturing but suffer from oxidative degradation, premature leakage during storage, opsonization-mediated clearance, and susceptibility to elevated temperatures, extreme pH, and high ionic strength (Obeid et al., 2023; Han et al., 2024; Liu et al., 2024; Yakubu et al., 2026). Polymeric nanoparticles (e.g., PLGA) enable tunable release, high drug loading, and superior gastrointestinal stability, yet they face synthetic complexity, limited industrial scalability under Good Manufacturing Practices (GMP) standards, suboptimal cellular internalization due to high negative surface charge, and incomplete long-term safety data (Liu et al., 2024; Tahvilian et al., 2026; Yakubu et al., 2026). Polymeric micelles effectively solubilize hydrophobic TCMs and are readily functionalized, yet they are limited by thermodynamic instability upon physiological dilution and the broader regulatory scarcity of approved injectable excipients for nanomedicines (Liu et al., 2024). SLNs/NLCs provide robust stability and BBB permeability, but the inherently low solubility of crystalline hydrophobic actives in solid lipid matrices as well as insufficient mechanistic understanding of cellular uptake and *in vivo* digestive processes pose limitations (Liu et al., 2024; Tahvilian et al., 2026). Nanoemulsions can achieve high encapsulation efficiency and enhanced gastrointestinal stability, yet they face thermodynamic instability (flocculation and Ostwald ripening), sterilization-induced degradation of labile payloads, and production cost alongside shelf-life instability, hindering industrialization (Han et al., 2024; Liu et al., 2024; Wahnou et al., 2025). Dendrimers offer monodisperse structures with tunable surface functionality and internal cavities for controlled drug loading, yet they remain academically confined due to high costs and poor scalability (Wahnou et al., 2025; Tahvilian et al., 2026). Inorganic and carbon-based carriers enable theranostics and exceptionally high drug loading, yet they remain clinically untranslatable due to non-biodegradability, reticuloendothelial accumulation with associated toxicity, and unresolved long-term safety concerns (Liu et al., 2024; Wahnou et al., 2025; Tahvilian et al., 2026). Protein-based carriers offer superior biocompatibility and receptor-mediated targeting potential, yet they face complex manufacturing and protein-specific aggregation-induced immunogenicity risks (Han et al., 2024; Liu et al., 2024). Exosomes exhibit inherently low immunogenicity and biomimetic capacity for biological barrier penetration; however, their clinical translation remains limited by the lack of standardized protocols, challenges in achieving scalable and reproducible isolation, and the early stage of translational development (Akter et al., 2025; Tahvilian et al., 2026). Critically, no existing platform simultaneously fulfills all the essential criteria—therapeutic payload compatibility, manufacturing scalability, pharmacokinetic stability, and regulatory feasibility—thereby necessitating a payload-driven selection strategy and the rational integration of hybrid design principles to bridge the gap between preclinical efficacy and clinical translation.

The studies reviewed span multiple cancer types and mechanisms (Tables 1–5), but breadth should not be confused with evidence strength. Most reports assess one formulation in one model without a free-compound control, carrier-only control, or common benchmark, making it difficult to separate nanocarrier effects from payload effects. Table 6, therefore, emphasizes not only the reported mechanisms and advantages but also missing pharmacokinetic, biodistribution, manufacturability, and long-term safety data. Recent phytochemical nanoformulations extend the range of proposed platforms, yet they do not resolve the same limitations of heterogeneous botanical composition and largely preclinical evidence (Danışman-Kalındemirtaş et al., 2024; Yazdanpanah et al., 2026; Bravo-Vázquez et al., 2026; Sandhya et al., 2026).

Despite encouraging preclinical activity, *in vivo* delivery remains constrained by tumor heterogeneity, variable EPR effects, and formulation-dependent pharmacokinetics. These interrelated factors limit extrapolation from cell culture or small-animal studies and are examined below.

### 5.1 Tumor heterogeneity, EPR variability, and personalized nanomedicine

The EPR effect is not a uniform property of all tumors. Between patients, and even among lesions in the same patient, NP delivery varies with vessel density and maturity, perfusion, endothelial transport, stromal fibrosis, interstitial fluid pressure, lymphatic drainage, tumor-associated macrophages, anatomical site, lesion size, and prior therapy. A meta-analysis found a median of only approximately 0.7% of the injected NP dose reaching solid tumors in animal studies, and human tumors generally show a broader and less predictable distribution (Wilhelm et al., 2016). Evidence that NPs can enter tumors through active transendothelial processes rather than passive leakage alone further argues against using EPR as a universal explanation (Sindhwani et al., 2020). Genetic and phenotypic heterogeneity also changes receptor abundance, endocytosis, metabolism, drug sensitivity, and immune composition; ligand targeting may, therefore, improve cellular uptake only in receptor-positive regions after the carrier has reached the lesion. Personalized nanomedicine should combine carrier design with patient and lesion stratification. Dynamic contrast-enhanced imaging, perfusion and vascular biomarkers, radiolabeled-carrier PET, or MRI-visible companion NPs can estimate delivery before treatment. In preclinical models, ferumoxytol MRI predicted the response to a therapeutic nanomedicine (Miller et al., 2015); in patients, ferumoxytol uptake correlated with response to nanoliposomal irinotecan, and 64Cu-MM-302 PET directly demonstrated marked interlesional variability in liposomal deposition (Ramanathan et al., 2017; Lee et al., 2017). Future trials of TCM nanoformulations should prospectively define delivery biomarkers, receptor eligibility, and adaptive dosing rather than enroll all patients under the assumption of a consistent EPR effect.

### 5.2 Quantitative pharmacokinetics, biodistribution, and clearance

Claims of improved bioavailability should be supported by formulation-specific comparisons. In healthy volunteers, two theracurmin products produced 18.4–20.5-fold higher Cmax and 35.9–42.6-fold higher AUClast than unformulated curcumin powder (Chung et al., 2021). However, higher plasma exposure does not prove tumor exposure. In a phase 1 study of intravenous liposomal curcumin in advanced cancer, the mean Cmax at 300 mg/m2 was 1,428 ng/mL over 6 h and 1,641 ng/mL over 8 h, yet plasma concentrations in most patients fell below quantification within 10 min after infusion, consistent with rapid redistribution and metabolism (Greil et al., 2018). These findings illustrate why AUC, Cmax, Tmax, terminal half-life, clearance, volume of distribution, and absolute or relative bioavailability should be reported together, with direct comparison to the free compound. Tissue biodistribution should quantify both carrier and pharmacologically active payload over time, including tumor-to-plasma and tumor-to-liver, spleen, and kidney ratios and intratumoral localization. Although Dey et al. (2022) included pharmacokinetic and biodistribution analyses for PLGA–diosgenin NPs, most studies summarized in Tables 1–6 did not provide comparable datasets. Particle size, shape, charge, rigidity, surface chemistry, and protein corona determine clearance. Very small particles may undergo renal filtration, whereas many larger or opsonized particles are sequestered by the mononuclear phagocyte system, historically termed the reticuloendothelial system, particularly the liver sinusoidal endothelial cells and Kupffer cells, with additional uptake by splenic macrophages (Choi et al., 2007; Tsoi et al., 2016). Such uptake can reduce the fraction available to tumors and create hepatic or splenic exposure, while poorly degradable inorganic materials may persist longer than lipid or polymer carriers. Future studies should distinguish intact carrier, free drug, and metabolites; report urinary and fecal excretion; and assess repeated-dose accumulation, immunogenicity, complement activation, and organ histopathology.

### 5.3 Clinical, regulatory, manufacturing, and economic barriers

Several clinical, regulatory, manufacturing, and economic barriers remain before TCM nanoformulations can be used routinely. These translational barriers are best exemplified by curcumin—the most extensively studied phytochemical nanomedicine—which, nonetheless, has yet to yield an approved oncology product despite extensive preclinical validation. This “curcumin paradox” illustrates three interconnected challenges relevant to the broader TCM nanomedicine field. First, clinical trial design faces inherent complexities in translating nanomedicine: while oral supplement-grade platforms predominate due to practical advantages in patient compliance, engineered injectable nanoparticles remain underrepresented. Moreover, establishing optimal dosing regimens requires meticulous dose-escalation and efficacy studies, and the identification of predictive biomarkers is crucial for patient stratification as not all individuals respond equally to these therapeutic approaches. The outcomes of trials such as CURTAXEL (NCT02095717) and SinaCurcumin (NCT02724618) indicate that neither simple oral supplementation nor nano-sizing alone can overcome intrinsic pharmacokinetic limitations or achieve therapeutic tumor accumulation without active targeting (ClinicalTrials.gov, 2026; Yakubu et al., 2026). Second, regulatory and manufacturing frameworks are inadequate for botanical nanomedicines: neither the FDA nor the EMA has issued dedicated guidance for NP-based botanical drugs, leaving developers to navigate stringent characterization requirements alongside raw material variability inherent to plant extracts (Tahvilian et al., 2026). Meanwhile, academic-scale synthesis methods lack GMP scalability, and terminal sterilization or lyophilization often triggers degradation and aggregation, creating a quality consistency gap that current quality-by-design (QbD) approaches have yet to fully bridge at the industrial scale. Third, commercial viability is constrained by structural economic and regulatory barriers rather than therapeutic potential alone. Platform economics vary markedly: liposomes offer medium cost with high scalability, whereas polymeric NPs and dendrimers entail high costs with moderate-to-low scalability. GMP compliance imposes substantial financial burdens, particularly for low- and middle-income countries, while inconsistent patent protection and consumer skepticism further weaken the business case. Structurally, many commercially marketed curcumin “nano” products (e.g., SinaCurcumin, Meriva, and Theracurmin) are sold as dietary supplements rather than approved oncology drugs. Consequently, formal cost-effectiveness analyses for TCM nanoformulations are limited, representing a critical gap in health economics research. Rather than replacing standard therapies, the most pragmatic translational pathway targets validated, high-need niches: (1) chemosensitization via ratiometric co-delivery; (2) supportive oncology (e.g., radiation dermatitis and cancer-related fatigue), where unmet needs and favorable risk–benefit profiles exist; and (3) long-term maintenance in low-risk populations, as exemplified by the phase 3 active-surveillance trial NCT03769766 (Yakubu et al., 2026). Crucially, multicenter head-to-head trials using carriers characterized by orthogonal analytical methods are urgently needed to establish quality benchmarks and regulatory precedents for the field.

### 5.4 Research priorities and future directions

Addressing these barriers requires coordinated priorities in quality control, targeting, model selection, safety, and clinical development. First, standardization and quality control should be prioritized to address the obstacles arising from the chemical complexity and natural variability of TCM extracts in consistent nanoformulation production (Hua et al., 2025). Subsequent research efforts should be directed toward establishing detailed standard operating procedures for the extraction, characterization, and nano-encapsulation of TCM components. For consistent batch production, efficient drug loading, and stable NPs, it is important to implement QbD principles along with advanced analytical methods (Zielińska et al., 2020; Marzi et al., 2022). AI-assisted design may support rational development of TCM-based nanoformulations, particularly when integrated with surface-enhanced Raman spectroscopy for fingerprint-level quality control and machine learning-driven QbD optimization. Such integration could improve fingerprint-based quality control and batch-to-batch reproducibility, but it requires prospective validation against conventional analytical workflows (Xia et al., 2025). Second, deeper mechanistic understanding and intelligent targeting are essential. Although many studies highlight anticancer effects, more in-depth investigations into the synergistic actions of multi-component TCM nanoformulations are necessary (Yang L. et al., 2024). Moreover, to enhance targeting specificity and overcome biological barriers, including the BBB, future advances in nanocarrier design should incorporate sophisticated stimuli-responsive elements, such as pH-labile, redox-sensitive, and enzyme-cleavable linkages, alongside multiligand targeting strategies (Rajankar et al., 2025; Ayyaril et al., 2023). Exploration of biomimetic NPs, including exosome-mimetic or cell membrane-coated nanocarriers, represents a promising avenue for improving biocompatibility and targeted delivery. Third, the rational design of combination therapies and immunomodulation is of significant importance. Future advancements in this area aim to develop well-characterized nanoplatforms for the co-delivery of TCM compounds alongside traditional chemotherapy, immunotherapy agents such as checkpoint inhibitors, or gasotherapy molecules such as carbon monoxide (Sun et al., 2025). This approach can harness synergistic effects to overcome MDR and potentiate antitumor immunity by promoting ICD and reversing the immunosuppressive tumor microenvironment. Studies should systematically assess these combinations utilizing robust *in vivo* models that accurately replicate the complexity of human tumors. As a specific implementation of these strategies, targeting cancer stem cells (CSCs) represents a promising therapeutic direction. Residual CSCs, endowed with self-renewal, drug-efflux capacity, and immune-evasive phenotypes, drive recurrence and metastasis. Nanocarriers can address them via CSC-biomarker-mediated active targeting (e.g., CD44/hyaluronic acid and CD133), niche remodeling, and co-delivery of anti-CSC TCM compounds with conventional agents (Duan et al., 2021). Notably, a nanovaccine integrating CSC-specific antigen display with epigenetic modulation of dendritic cells simultaneously eradicated CSCs and bulk tumors, suppressing postoperative recurrence (You et al., 2025). However, CSC heterogeneity, marker nonspecificity, and the lack of standardized evaluation models remain key translational hurdles. Fourth, it is essential to focus on a thorough safety evaluation and clinical translation. A significant translational gap persists between promising preclinical findings and clinical application, underscoring the need for comprehensive investigations into the long-term pharmacokinetics, biodistribution, potential off-target effects, and biodegradation of these nanoformulations (Hnamte and Pulikkal, 2024). Large-scale GMP-compliant manufacturing processes must be developed to enable clinical-grade production. Ultimately, robust clinical trials with rigorous design are indispensable to demonstrate the safety, efficacy, and potential therapeutic value of TCM nanoformulations compared to current standards of care. Such validation is necessary to facilitate their integration into conventional oncology practice.
